# Tuning magnetocrystalline anisotropy by controlling the orbital electronic configuration of two-dimensional magnetic materials

**DOI:** 10.1039/d3na00003f

**Published:** 2023-03-28

**Authors:** Xiaoxiao Guan, Yun Zhang, Xia Long, Guo-Jun Zhu, Juexian Cao

**Affiliations:** a Department of Physics, Hunan Institute of Advanced Sensing and Information Technology, Xiangtan University Hunan 411105 China gjzhu17@fudan.edu.cn jxcao@xtu.edu.cn; b Department of Physics and Information Technology, Baoji University of Arts and Sciences Baoji 721016 China; c Key Laboratory for Computational Physical Sciences (MOE), State Key Laboratory of Surface Physics, Department of Physics, Fudan University Shanghai 200433 China

## Abstract

A suitable magnetic anisotropy energy (MAE) is a key factor for magnetic materials. However, an effective MAE control method has not yet been achieved. In this study, we propose a novel strategy to manipulate MAE by rearranging the d-orbitals of metal atoms with oxygen functionalized metallophthalocyanine (MPc) by first-principles calculations. By the dual regulation of electric field and atomic adsorption, we have achieved a substantial amplification of the single regulation method. The use of O atoms to modify the metallophthalocyanine (MPc) sheets effectively adjusts the orbital arrangement of the electronic configuration in the d-orbitals of the transition metal near the Fermi level, thereby modulating the MAE of the structure. More importantly, the electric field amplifies the effect of electric-field regulation by adjusting the distance between the O atom and metal atom. Our results demonstrate a new approach to modulating the MAE of two-dimensional magnetic films for practical application in information storage.

## Abstract 2

The using of O atoms to modify metallophthalocyanine sheets effectively adjusts the orbital arrangement of the electronic configuration of d-orbitals in transition metals, thereby modulating the MAE of the structure.

## Graphical abstract

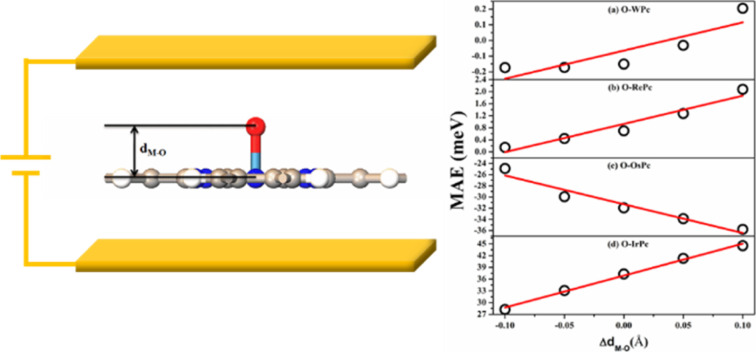

## Introduction

Studies in the field of magnetocrystalline anisotropy (MCA) are extremely vital for various applications, such as permanent magnets and magnetic data storage.^1–4^ Reducing the size and stabilizing the magnetic order of nanoparticles are the key issues to improving their magnetic storage density at room temperature. Recently, many two-dimensional magnetic materials have been reported with properties suitable for applications in various fields, such as electronic devices, optoelectronic devices, and catalysts.^5–10^ Similar to three-dimensional (3D) materials, two-dimensional (2D) magnetic materials should also have appropriate MAE for them to be used. Due to the broken symmetry in the vertical direction, 2D materials tend to exhibit better MCA. A great experimental breakthrough was made in 2D ferromagnetic materials when periodic Fe-functionalized phthalocyanine networks were synthesized experimentally,^11^ which proved to be promising materials for future spintronic applications due to their sizable MAE and versatile nanostructures.^11,12^ Unlike the recently developed two-dimensional magnetic materials, such as CrI_3_ (ref. 13 and 14) and Cr_2_Ge_2_Te_6_,^15^ the Fe-functionalized phthalocyanine network is composed of an organic framework, and the transition metal atoms in the center can be easily replaced with other metal elements. This means that the material can have highly varied and rich properties.

In ordinary ferromagnetic materials, a reduction in size leads to a decrease in the magnetocrystalline anisotropy energy (MAE) and results in superparamagnetic relaxation at nanoscopic sizes. An emerging problem lies in increasing the MAE of the recording unit to inhibit magnetization reversal induced by thermal fluctuations.^16–18^ For practical application at room temperature, it is crucial to find magnetic nanostructures with MAE up to 30–50 meV. Identifying ways to effectively enhance the range of MAE controlled by the applied method is an important topic of research in spintronics. As per the second-order perturbation theory,^19,20^ it is obvious that increasing the strength of spin–orbit coupling (SOC) and adjusting the energy level difference between the occupied state and the non-occupied state will be effective methods of modulating MAE. The strength of SOC is difficult to control after material synthesis. Therefore, modulating the energy level position is the most convenient method in practice as it is an important indicator of the MAE of magnetic materials used for practical applications. It has been demonstrated that MAE can be modulated by means of thermal activation,^21,22^ external strain,^23–25^ or external electric field.^24,26,27^ However, single control methods often face the problem of a narrow adjustment range.

In this paper, we propose an alternative and novel route for the modulation MAE of low-dimensional nanostructures by first-principles calculations. The key development in this work is the rearrangement of the d-orbital configuration of transition metals by combining the effect of the electric field with oxygen functionalization. We could amplify the amplitude of pure electric field modulation by changing the interatomic distance under the action of the electric field. By taking the oxygen-functionalized metallophthalocyanine (MPc) network as the prototype, we show that MAE dramatically changes with the variation in bond length between the oxygen and transition metal atoms. The changes in MAE can be attributed to the rearrangements of the electronic configuration in the d-orbital of the transition metals induced by the regulation of the interaction between oxygen and the metals. With the functionalization of oxygen atoms, the effect of the electric field is amplified by adjusting the distance between the O atoms and metal atoms. Our study provides an innovative approach for designing a nanoscale magnetic storage medium for experimental exploration and practical applications.

## Calculation methods

All our first-principles calculations were performed based on the density functional theory (DFT) as implemented in the Vienna *ab initio* simulation package (VASP).^28,29^ We used the projector augmented wave (PAW) method^30,31^ to describe the interaction between the valence electrons and the ionic cores. The spin-polarized generalized gradient approximation (GGA) prescribed by Perdew–Burke–Ernzerhof (PBE)^32^ was used for calculating the exchange-correlation potential. The vacuum thickness along the *z*-axis was set to 20 Å, which is large enough to avoid interactions between the nearest neighboring images. A kinetic energy cutoff of 500 eV was used for plane-wave basis expansion. We used a sigma of 0.01 eV with the Gaussian smearing method to describe the occupation. For the Brillouin zone sample, the reciprocal space was presented by the Monkhorst–Pack special *k*-points method^33^ with a 6 × 6 × 1 mesh for geometry relaxation and a 13 × 13 × 1 mesh without symmetry constraints for static calculations. The convergence criteria were set to 1 × 10^−6^ eV and 1 × 10^−2^ eV Å^−1^ for total energy and Hellman–Feynman forces on each atom, respectively. The MAE was determined by the torque approach.^19,20^

To further discuss the MAE modifications, we noted that the MAE is well related to the electronic configuration of the spin orbitals through second-order perturbation. The contributions of MAE are subdivided into MAE^uu^, MAE^dd^ and MAE^ud,du^, among which MAE^uu^ and MAE^dd^ denote the contributions from the same spin channel, and MAE^ud,du^ denotes the contribution from the crossover spin channels. According to the second-order perturbation theory, the same spin channel contributions can be expressed as1
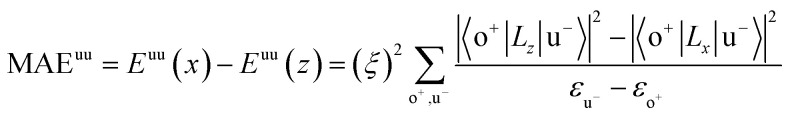
where o and u denote the occupied and unoccupied electronic states, respectively. *ε*_o^+^_ and *ε*_u^−^_ are their corresponding occupied majority-spin and unoccupied minority-spin states, respectively. Moreover, *L*_*z*_ and *L*_*x*_ are the angular momentum operators. *ξ* is the strength of SOC. Similarly, the contributions from the crossover spin channels to MAE can be expressed as2
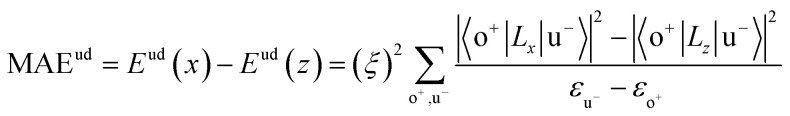


For contributions from the d states of the transition metals, the nonzero matrix elements of the *L*_*z*_ and *L*_*x*_ operators are 〈*xz*|*L*_*z*_|*yz*〉 = 1, 〈*x*^2^ − *y*^2^|*L*_*z*_|*xy*〉 = 2, 〈*xy*|*L*_*x*_|*xz*〉 = 1, 〈*x*^2^ − *y*^2^|*L*_*x*_|*yz*〉 = 1 and 
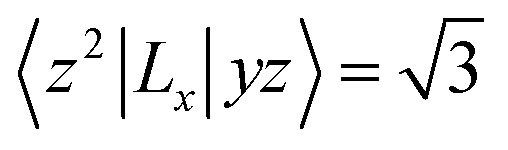
. According to eqn (1), the positive contributions to MAE originate from two matrix elements 〈*xz*|*L*_*z*_|*yz*〉 and 〈*x*^2^ − *y*^2^|*L*_*z*_|*xy*〉, while 〈*xy*|*L*_*x*_|*xz*〉, 〈*x*^2^ − *y*^2^|*L*_*x*_|*yz*〉 and 〈*x*^2^ − *y*^2^|*L*_*x*_|*yz*〉 denote negative contributions. However, for coupling between crossover spin channels, it is just the opposite of the case described in eqn (2). Obviously, the best modulation method is changing the orbital electronic configuration to increase the contributions we want. The most convenient approach is introducing an atom to the functional metal atom for controlling the orbital electronic configuration.

## Results and discussion

**Fig. 1:**
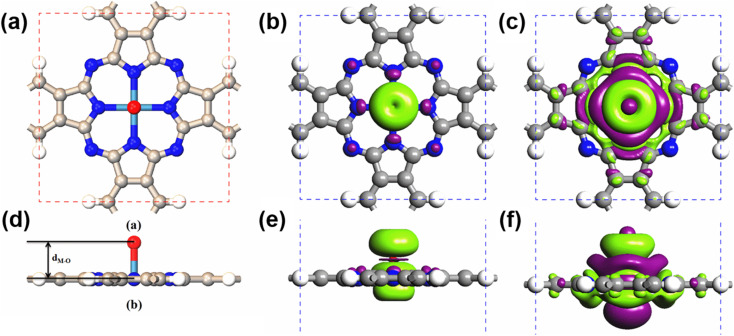
The top view and side view of the (a) and (d) atomic arrangement, (b) and (e) charge density difference and (c) and (f) spin density of the oxygen-functionalized MPc network. The white, gray, blue, light blue and red spheres represent the H atom, C atom, N atom, metal atom and O atom, respectively. The purple and green isosurfaces indicate charge depletion and accumulation, respectively. Spin polarization is predominantly around the metal and the above oxygen atom, in conjunction with weakly induced spin polarization around the N and C atoms.

Due to their large SOCs, we chose 5d-metal-functionalized Pc networks as our initial structures. Since oxygen is a common doping element, we investigated oxygen functionalized 5d transition-metal doped Pc networks. As typical examples, here, we took four transition metals, namely W, Re, Os and Ir into consideration. As shown in Fig. 1, the 2D MPc network belonged to the *P*4/*mmm* symmetry group. The metal atom was located at the center of the unit cell and surrounded by four nitrogen atoms. In the atomic arrangement of the oxygen-functionalized MPc network, an additional oxygen is adsorbed on top of the metal atom in each unit cell of the MPc network. All atomic positions of the oxygen-functionalized MPc networks involving their lattice constants are fully relaxed. The optimized lattice constants and the bond lengths between the metal atom and the neighboring oxygen *d*_M–O_ are given in Table 1. It was found that the lattice constants of the oxygen-functionalized MPc networks considered are in the range of 10.75 ± 0.05 Å, which is similar to the lattice constant of intrinsic MPc networks reported in previous studies.^22^ The bond length between oxygen and metal *d*_M–O_ ranged from 1.721 to 1.853 Å.

**Table 1:** The lattice constants *a* (in Å), bond lengths between the metal atoms and the O atom *d*_M–O_ (in Å), total magnetic moments Mag (in *μ*_B_), and the magnetocrystalline anisotropy energy per unit cell MAE (in meV) of the metal-functionalized Pc networks (MPc, M = W, Re, Os and Ir) with or without oxygen functionalization

System	*a* (Å)	*d*_O–M_ (Å)	Mag (*μ*_B_)		MAE (meV)	
			With O	Without O	With O	Without O
WPc	10.793	1.725	1.13	3.55	−0.15	17.72
RePc	10.772	1.721	0.15	2.99	0.70	20.25
OsPc	10.727	1.781	2.00	1.02	−31.95	−12.67
IrPc	10.720	1.853	0.96	0.62	37.31	−5.92

**Fig. 2:**
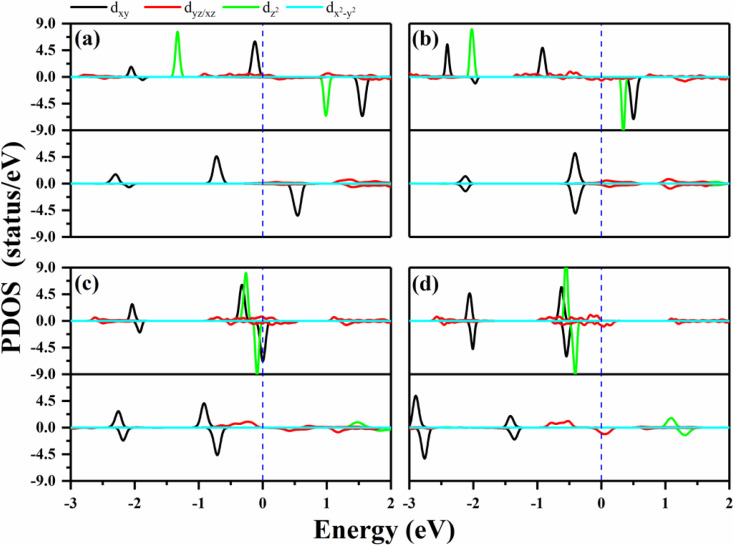
The PDOS of the metals in the 2D MPc networks with and without oxygen functionalization. (a)–(d) Represent WPc, RePc, OsPc and IrPc, respectively. The upper panels present the PDOS of the metals in MPc, and the lower panels show the PDOS of metals in the oxygen-functionalized MPC. Positive and negative values for PDOS stand for the majority and minority spin channels, respectively. The blue dashed lines indicate the Fermi level.

After obtaining the optimized structure, the magnetic moments of the oxygen-functionalized MPc (M = W, Re, Os and Ir) networks were calculated and are shown in Table 1. For comparison, the magnetic moments of the intrinsic MPc networks are also shown. After the O modification, the magnetic moment changed considerably. This change means that the O atom and the metal atom have a strong hybridization. In general, the total magnetic moments of MPc networks follow the rule of 8 − *n μ*_B_ due to the *P*4/*mmm* symmetry, where *n* is the d electron number of the transition metal. However, the total magnetic moments of 5d transition-metal-doped Pc networks slightly decrease due to the strong hybridization between the transition metal and nitrogen atoms. Herein, the magnetic moments of intrinsic WPc, RePc, OsPc and IrPc were 3.55, 2.99, 1.02 and 0.62 *μ*_B_, respectively. Similar to MPc networks and MPc molecules, the magnetic moments of the oxygen-functionalized MPc networks originate from the transition metal at the center. It is clear from the spin density shown in Fig. 1(b) that spin polarization was predominantly around the metal and the above oxygen atom, in conjunction with weakly induced spin polarization around the N and C atoms. However, the magnetic moment of an oxygen-functionalized MPc is greatly influenced by the strong hybridization between the O–p_*z*_ and the Metal–d_*z*^2^_ orbitals. To illustrate the charge density redistribution after oxygen adsorption on the MPc network, we have given the charge difference for oxygen-functionalized OsPc as a typical example. Here, the charge difference was defined as Δ*ρ* = *ρ*_O–MPc_ − *ρ*_MPc_ − *ρ*_O_, where *ρ*_O–MPc_, *ρ*_MPc_, and *ρ*_O_ are the total charge densities of the oxygen-functionalized MPc network, the intrinsic MPc network and the adsorbed oxygen atom, respectively. Obviously, much of the charge density depletes from the Os–d_*z*^2^_ orbital and is transferred to the adsorbed oxygen atom, leading to a reduction in the local magnetic moment of the metal atom in the oxygen-functionalized MPc networks, as per the charge difference shown in Fig. 2(b). This results in the total magnet moments of 1.13, 0.15, 2.00 and 0.96 *μ*_B_ for the O–WPc, O–RePc, O–OsPc and O–IrPc networks, respectively, as shown in Table 1.

In order to verify the orbital configuration, we evaluated the orbital projected density of states (PDOS) for the transition metals in the O–MPc networks with M = W, Re, Os and Ir, as shown in Fig. 2. For comparison, the PDOS of the metals in intrinsic MPc networks are also shown. Due to the *P*4/*mmm* symmetry, the five d-orbitals of the metal atoms split into four energy levels. Typically, the energy sequence from the lowest to highest of the d-states of the transition metal are assigned as *xy*, *xz*/*yz*, *z*^2^ and *x*^2^ − *y*^2^, similar to those of their orbitals in transition-metal-doped Pc molecules (MPc). However, the actual electronic occupations of these orbitals shown in Fig. 2 are somewhat different from this simple assignment due to the strong hybridization between the transition metals and their neighboring N atoms. From the PDOS of the intrinsic MPc networks shown in Fig. 2 (upper panels), one can find that the *x*^2^ − *y*^2^ orbital remains unoccupied in both spin channels, as shown by the light blue curves in Fig. 2, which means that the *x*^2^ − *y*^2^ orbital loses charges to the N–2p states during the formation of MPc. The *xy* and *xz*/*yz* states strongly hybrid with the N–2p states and split into several peaks. The nonbonding *z*^2^ orbital is located near the Fermi level with one electron occupation in the majority spin channel in the cases of WPc and RePc, while the *z*^2^ orbitals of Os and Ir are fully occupied both in the majority spin channel and minority spin channel, as shown in Fig. 2(c) and (d). After functionalized with oxygen, the *z*^2^ orbitals of the transition metals lose their charges to the O–p_*z*_ orbital and are located about 2 eV above the Fermi level due to the strong hybridization of the metals and oxygen. The strong hybridization modifies the electronic configuration of the d orbitals of the transition metal, resulting in variation in the magnetic moment of the oxygen-functionalized MPc.

The MAEs of oxygen-functionalized MPc networks, as well as those of intrinsic MPc networks, were calculated, as shown in Table 1. It was found that the MAEs of the intrinsic WPc, RePc, OsPc and IrPc were 17.72, 20.25, −12.67 and −5.92 meV, respectively. Here, the positive and negative values indicate that the easy axis of spin orientation is out-of-plane and in-plane, respectively. Interestingly, the MAEs were greatly modified by decoration with oxygen and even involved the transition of the easy axis from being in-plane to out-of-plane or the inverse. For example, the intrinsic IrPc network had an MAE of −5.92 meV with an in-plane easy axis, while the MAE enhanced up to 37.31 meV with an out-of-plane easy axis after functionalization with oxygen, as shown in Table 1. The enhancement of the MAE of IrPc originates from the rearrangement of the electronic configuration in the d orbitals of the transition metal, as discussed above. For example, the negative contributions to the MAE of IrPc originate from the matrix element 〈*xz*|*L*_*z*_|*yz*〉 in eqn (2) with crossover spin channels. Meanwhile, the very small negative contribution of O–IrPc comes from 〈*xy*|*L*_*x*_|*xz*〉 in eqn (1) with the same spin channels and 〈*z*^2^|*L*_*x*_|*yz*〉 with crossover spin channels.

**Fig. 3:**
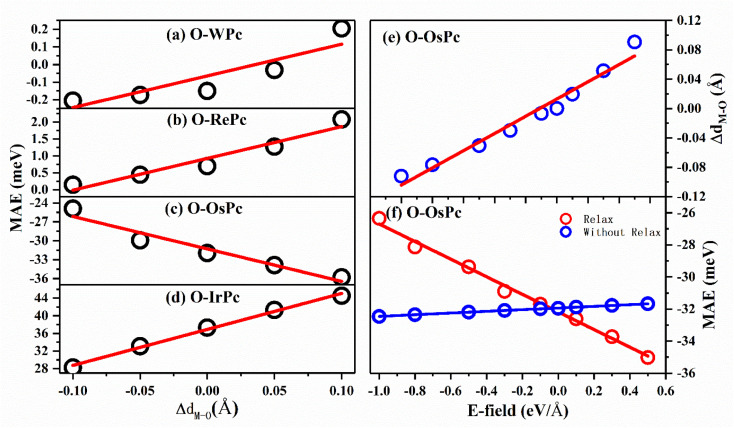
The magnetic anisotropy energy for oxygen functionalized (a) WPc; (b) RePc; (c) OsPc and (d) IrPc with different vertical position of oxygen on the top of metal atom. The preferable position of oxygen is taken as the reference. (e) The distance and (f) MAE of O–OsPc as a function of the electric field.

This inspired us to speculate that MAE can be easily modulated by controlling the electronic configuration in the d orbitals of the transition metals in MPc networks. This can be realized by changing the interaction between the oxygen and metal atoms. The MAEs of oxygen-functionalized MPc with different M–O bond lengths are shown in Fig. 3. We took the bond length of M–O in the preferable structure as the reference. Huge changes in MAE were found when the variation in bond length *d*_M–O_ ranged from −0.1 to 0.1 Å. Especially, it was found that the MAE of the IrPc network was almost enhanced by 60% when the bond length *d*_Ir–O_ was increased only by 0.1 Å. Unlike other structures, the trend of the MAE of O–OsPc was different, as shown in Fig. 3(c). The stretched *d*_O–M_ reduced the denominator in eqn (1) and enhanced the 〈*xy*|*L*_*x*_|*xz*〉 effect, causing MAE to increase in the negative direction. More interestingly, the MAE of O–WPc was not only greatly enhanced but also involved a transition of the easy axis from in-plane to out-of-plane when the W–O bond length was stretched, as shown in Fig. 3(a).

Our results demonstrate a novel approach to modulating the MAE of oxygen-functionalized MPc networks.

**Fig. 4:**
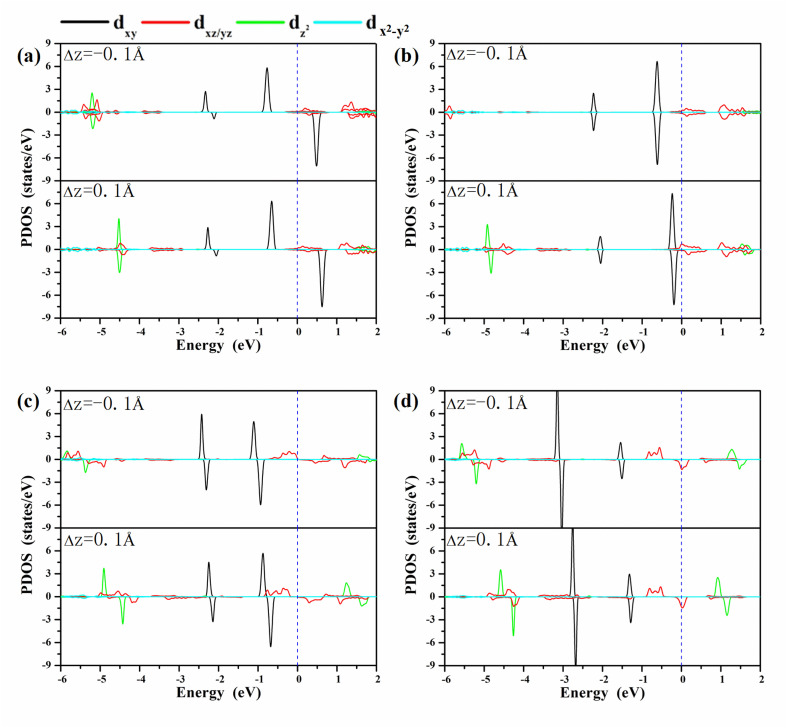
The PDOS of the metals in the oxygen-functionalized MPc networks with different positions of oxygen. (a) O–WPc; (b) O–RePc; (c) O–OsPc; (d) O–IrPc. The positive and negative values of the PDOS stand for the majority and minority spin channels, respectively. The blue dashed line indicates the Fermi level. The height of the O atoms can effectively adjust the d orbital arrangement of the metal atoms.

A clear understanding of the relationship between MAE and the bond length *d*_O–M_ is imperative to guide the development of a new strategy to modulate the MAE. We investigated the PDOS of the metals in the oxygen-functionalized MPc with Δ*d*_M−O_ = ±0.1 Å, as shown in Fig. 4. The most important common feature was that the energy levels of the d-orbitals, especially the d_*xy*_ orbital, shifted upward to the Fermi level when the bond length *d*_M–O_ was stretched. The shift in energy level plays an important role in changing the MAE according to eqn (1) and (2). In general, the negative contribution to MAE is provided by the coupling between the occupied d_*xz*_ (d_*yz*_) and unoccupied d_*yz*_ (d_*xz*_) states through the matrix elements of the angular momentum operator *L*_*z*_, while the positive MAE contribution is from the spin–orbit coupling interaction between the occupied minority spin d_*xy*_ and the unoccupied majority spin d_*xz*_/d_*yz*_. Obviously, the energy difference between the unoccupied states and occupied states is greatly reduced when the bond length *d*_M–O_ is stretched, resulting in a huge variation in the MAE of the oxygen-functionalized MPc.

Up to now, regulating the interaction between oxygen and metal atoms has been the key point in the practical application of magnetic storage devices. Due to the polarization effect, the interaction between oxygen and metal can be easily controlled by applying an external electric field. Taking the oxygen-functionalized OsPc network as a typical example, the difference in the bond length Δ*d*_Os–O_ observed under an external electric field is shown in Fig. 3(e). It was found that the bond length *d*_Os–O_ almost linearly increased with the increasing strength of the external electric field. Especially, the Δ*d*_Os–O_ value reached 0.1 Å under an electric field of about 0.5 eV Å^−1^, while the bond length *d*_Os–O_ shrunk by about 0.1 Å when the applied electric field was about −1.0 eV Å^−1^. Here, the negative and positive values indicate the orientation of the external electric field. Our results clearly demonstrate that the interaction between the oxygen and metal atoms can be effectively controlled by the external electric field.

More importantly, with the change in the distance between the O atom and the metal atom under the action of the electric field, the MAE range controlled by the electric field in the system has been greatly expanded. As shown in Fig. 3(f), when the movement of the oxygen atom position under the electric field was not considered, the regulation of the MAE of the system by the electric field was only up to 2 meV; however, the regulation of the electric field could be greatly enhanced up to 10 meV after considering atomic relaxation. This means that the introduction of oxygen atoms can greatly enhance the controlling effect of the electric field on the MAE of the system through interatomic distance modulation.

## Conclusion

In conclusion, in this work, we have demonstrated a novel approach to modulate the MAE of magnetic materials by controlling the electronic configuration in the d orbital of transition metals through first-principles calculations. By combining electric field and atomic doping, we have achieved continuous and large-scale tuning of the MAE. As typical examples, we have applied the method to modulate the MAEs of oxygen-functionalized MPc networks. Our results show that the MAEs of the oxygen-functionalized MPc are greatly enhanced, especially those of the oxygen-functionalized IrPc networks, when the bond length *d*_M–O_ is stretched as the interaction enhances. More importantly, the variation in the atomic distance under the electric field greatly amplifies the range of MAE controlled by the electric field. We hope that this work will stimulate experimental interest and look forward to validating our theoretical prediction.

## Author contributions

Guo-Jun Zhu and Juexian Cao conceived the ideas, Guo-Jun Zhu, Xiaoxiao Guan, Juexian Cao and Yun Zhang analyzed the data, Guo-Jun Zhu, Xiaoxiao Guan and Juexian Cao wrote the paper, Guo-Jun Zhu, Xiaoxiao Guan and Xia Long performed the calculations.

## Conflicts of interest

The authors declare no competing interests.

## Supplementary Material

## References

[cit1] Dieny B., Chshiev M. (2017). Rev. Mod. Phys..

[cit2] Wang Y., Li X., Zheng X., Yang J. (2018). Phys. Chem. Chem. Phys..

[cit3] Abramchuk M., Jaszewski S., Metz K. R., Osterhoudt G. B., Wang Y., Burch K. S., Tafti F. (2018). Adv. Mater..

[cit4] Geng J., Chan I. N., Ai H., Lo K. H., Kawazoe Y., Ng K. W., Pan H. (2020). Phys. Chem. Chem. Phys..

[cit5] Wu Z., Yu J., Yuan S. (2019). Phys. Chem. Chem. Phys..

[cit6] Xu Q.-F., Xie W.-Q., Lu Z.-W., Zhao Y.-J. (2020). Phys. Lett. A.

[cit7] Lei C., Chittari B. L., Nomura K., Banerjee N., Jung J., MacDonald A. H. (2021). Nano Lett..

[cit8] Zhang Y., Holder T., Ishizuka H., de Juan F., Nagaosa N., Felser C., Yan B. (2019). Nat. Commun..

[cit9] Sakamoto S. (2022). *et al.*. Phys. Rev. B.

[cit10] Sakamoto S., Nozaki T., Yuasa S., Amemiya K., Miwa S. (2022). Phys. Rev. B.

[cit11] Abel M., Clair S., Ourdjini O., Mossoyan M., Porte L. (2011). J. Am. Chem. Soc..

[cit12] Wang J., Shi Y., Cao J., Wu R. (2009). Appl. Phys. Lett..

[cit13] Seyler K. L. (2018). *et al.*. Nat. Phys..

[cit14] Liu J., Sun Q., Kawazoe Y., Jena P. (2016). Phys. Chem. Chem. Phys..

[cit15] Xu C., Feng J., Xiang H., Bellaiche L. (2018). npj Comput. Mater..

[cit16] Gambardella S. R. P., Veronese M., Dhesi S. S., Grazioli C., Dallmeyer A., Cabria I., Zeller R., Dederichs P. H., Kern K., Carbone C., Brune H. (2003). Science.

[cit17] Hu J., Wu R. (2014). Nano Lett..

[cit18] Strandberg T. O., Canali C. M., MacDonald A. H. (2007). Nat. Mater..

[cit19] Wu R., Freeman A. J. (1999). J. Magn. Magn. Mater..

[cit20] Wang X., Wu R., Wang D., Freeman A. J. (1996). Phys. Rev. B: Condens. Matter Mater. Phys..

[cit21] El-Gendy A. A., Bertino M., Clifford D., Qian M., Khanna S. N., Carpenter E. E. (2015). Appl. Phys. Lett..

[cit22] Zhu G., Zhang Y., Xiao H., Cao J. (2016). J. Solid State Chem..

[cit23] Zhou J., Wang Q., Sun Q., Kawazoe Y., Jena P. (2012). J. Phys. Chem. Lett..

[cit24] Zhou J., Wang Q., Sun Q., Kawazoe Y., Jena P. (2015). Phys. Chem. Chem. Phys..

[cit25] Gao T. (2017). *et al.*. Nano Lett..

[cit26] Hu J., Wu R. (2013). Phys. Rev. Lett..

[cit27] Negulyaev N. N., Stepanyuk V. S., Hergert W., Kirschner J. (2011). Phys. Rev. Lett..

[cit28] Kresse G., Furthmüller J. (1996). Comput. Mater. Sci..

[cit29] Kresse G., Furthmüller J. (1996). Phys. Rev. B: Condens. Matter Mater. Phys..

[cit30] Blöchl P. E. (1994). Phys. Rev. B: Condens. Matter Mater. Phys..

[cit31] Kresse G., Furthmüller J. (1996). Phys. Rev. B: Condens. Matter Mater. Phys..

[cit32] Perdew J. P., Burke K., Ernzerhof M. (1996). Phys. Rev. Lett..

[cit33] Monkhorst H. J., Pack J. D. (1976). Phys. Rev. B: Solid State.

